# Semi-Supervised Variational Autoencoders for Out-of-Distribution Generation

**DOI:** 10.3390/e25121659

**Published:** 2023-12-14

**Authors:** Frantzeska Lavda, Alexandros Kalousis

**Affiliations:** 1 Geneva School of Business Administration (DMML Group), University of Applied Sciences and Arts Western Switzerland (HES-SO), 1227 Geneva, Switzerland; alexandros.kalousis@hesge.ch; 2 Faculty of Science, Computer Science Department, University of Geneva, 1214 Geneva, Switzerland

**Keywords:** generative models, VAE, back translation

## Abstract

Humans are able to quickly adapt to new situations, learn effectively with limited data, and create unique combinations of basic concepts. In contrast, generalizing out-of-distribution (OOD) data and achieving combinatorial generalizations are fundamental challenges for machine learning models. Moreover, obtaining high-quality labeled examples can be very time-consuming and expensive, particularly when specialized skills are required for labeling. To address these issues, we propose BtVAE, a method that utilizes conditional VAE models to achieve combinatorial generalization in certain scenarios and consequently to generate out-of-distribution (OOD) data in a semi-supervised manner. Unlike previous approaches that use new factors of variation during testing, our method uses only existing attributes from the training data but in ways that were not seen during training (e.g., small objects of a specific shape during training and large objects of the same shape during testing).

## 1. Introduction

Combinatorial generalization, the ability to understand and produce novel combinations of familiar elements, is a key aspect of human intelligence. Humans can make “infinite use of finite means” [1,2], using a small set of elements (such as words) to create limitless combinations (such as new sentences) [3]. For example, one can imagine a pink elephant even if they have never seen one before. While color and object are independent, for a human brain imagining a pink elephant is a trivial task. However, it is not as straightforward for machine learning (ML) models to generate a pink elephant if there are no pink elephants in the training data as they struggle with generating out-of-distribution (OOD) data or mixing existing attributes (color and object) [4,5,6,7]. The authors of [3] argue that combinatorial generalization should be one of the top priorities in modern artificial intelligence.

Given the fact that acquiring high-quality labeled examples can be time-consuming and expensive, various semi-supervised learning (SSL) algorithms have been proposed for deep learning [8,9,10,11,12], generative modeling [13,14,15], and disentanglement learning [13,14,15,16]. These learning algorithms attempt to efficiently utilize the large number of unlabeled data alongside a relatively small number of labeled data. However, the performance of the SSL algorithms can degrade when the unlabeled data contain OOD examples [17] since SSL methods are designed with the assumption that both unlabeled and test data come from the same distribution as the labeled data.

OOD generation is a relatively new field of research. Data augmentation, known for its effectiveness in enhancing data diversity, plays a pivotal role in improving OOD generalization capabilities [18]. Studies such as [7,19,20,21,22] have utilized generative models like GANs and VAEs to produce OOD samples in order to improve the efficacy of OOD detection. While their focus is not on the model’s capability to generate OOD data, they augment the training set with generated samples to improve the learning process of OOD classifiers, with the ultimate goal of detecting OOD inputs. The OOD samples generated in these studies typically either mimic the characteristics of in-distribution data or are restricted to the periphery of the in-distribution data space. In contrast, our approach diverges significantly from these methods as our objective is not OOD detection. Moreover, our method aims to generate OOD data that do not mimic the characteristics of in-distribution data but have desired OOD properties. Rather than generating samples from the boundary of in-distribution data, we concentrate on the generation of OOD samples with desired attribute value combinations that do not exist in the in-distribution data.

Moreover, recent advancements in domain adaptation leverage the capabilities of generative models [23,24,25,26], focusing on adapting pre-trained generators to create images for new target domains, OOD. The objective here is to effectively transfer and adapt the variable factors learned from in-distribution data to OOD contexts. However, a notable limitation of these adapted models is their tendency to lose the capability to generate samples from the original in-distribution data post-adaptation. In contrast to domain adaptation approaches, our aim is to generate OOD data while preserving the model’s original functionality with in-distribution data. This approach ensures a more versatile model capable of handling both in-distribution and OOD data effectively.

Variational Autoencoders (VAEs) have demonstrated remarkable versatility, with applications across a diverse range of domains. These include drug design [27,28], image retrieval [29,30,31], and dialog generation [32,33]. While VAEs are effective in specific scenarios, they often struggle to combine attributes in ways that reflect human cognitive processes. This limitation becomes more pronounced in situations where training data lack diversity or when the model encounters novel combinations of attribute values. This gap highlights the necessity for more sophisticated generative models capable of addressing these challenges. For instance, Ref. [34], a VAE, combined with image processing techniques, was applied in game design to successfully generate new game maps. However, our approach diverges as we aim to generate new samples that encompass desired OOD attribute value combinations, thereby expanding what VAEs can achieve in terms of data generation and diversity.

Recently, several papers used OOD datasets similar to those used in this paper to explore whether models that have high disentanglement performance are also able to perform certain forms of combinatorial generalization [35,36,37,38,39,40]. Refs. [37,41] showed promising results using disentanglement models for OOD tasks, but the models were tested on simple OOD data, where only a small number of combinations were excluded [38]. In contrast, recent studies, such as [38,39,40], tested different models under more challenging conditions and found no evidence that the disentanglement representation supports combinatorial generalization, in both latent space and reconstruction space, under challenging generalization conditions (where a larger number of combinations are excluded from the training set). In this paper, we test the OOD generation performance of our model by excluding specific combinations of attribute values from the training data, similarly to the approach employed in [38,39,40]. However, our objective diverges from aiming for a disentangled latent space; instead, we focus on the conditional generation of unseen property combinations.

In response to these challenges, we propose BtVAE (Back-translation Variational Autoencoder), a semi-supervised generative model that leverages the strengths of conditional VAEs while incorporating a back-translation procedure to improve their ability to handle OOD data. We aim to address the challenge of reconstructing out-of-distribution data as well as generating samples that exhibit unseen combinations of attributes values. Our evaluation of BtVAE’s performance on multiple datasets highlights its ability to reconstruct and generate OOD samples with impressive accuracy. Moreover, we show BtVAE’s capability to effectively manipulate OOD data by changing the attributes according to the desired values.

The main contributions of our work include:

1. We propose BtVAE, a semi-supervised approach for generating and reconstructing OOD data based on previously unseen combinations of attribute values. Our method leverages conditional VAEs to achieve combinatorial generalization.
2. We evaluate the performance of BtVAE on various datasets and demonstrate its effectiveness in reconstructing and generating OOD samples with high fidelity, even when the available labeled data are limited.
3. We show that BtVAE can effectively manipulate the OOD data by changing the attributes according to the desired values, demonstrating its potential for various real-world applications where new combinations of attribute values might emerge.

The remainder of the paper is organized as follows: In Section 2, we provide a background on VAEs and semi-supervised learning. Section 3 describes the proposed BtVAE method in detail. In Section 4, we present the experimental results and discuss the performance of BtVAE in various datasets. Finally, Section 5 concludes the paper and outlines possible directions for future research.

## 2. Background

### 2.1. Semi-Supervised Learning

Semi-supervised learning (SSL) combines a small number of labeled data with a large number of unlabeled data during training. The underlying assumption is that the structure of the data can be learned from the unlabeled samples, while the labeled data provide guidance for accurate prediction [42]. SSL algorithms are particularly useful when labeled data are scarce or expensive to acquire, which is often the case in real-world applications. Various SSL algorithms have been proposed for deep learning, such as consistency regularization [10,11], mixup-based methods [8,9], pseudo-labeling [8,13,43] and teacher-student models [12]. These algorithms generally rely on the assumption that test data and unlabeled data originate from the same distribution as labeled data. Each of these methods is characterized by a unique loss function, reflecting its core principle and approach to leverage both labeled and unlabeled data.

**Consistency Regularization:**

Consistency regularization relies on the idea that the model should output similar predictions for an unlabeled data point and its perturbed version. This can be mathematically represented as:

$$L_{\mathrm{consistency}}=E_{x∼U,\tilde{x}∼T\left(x\right)}\left[{\left|f\left(x\right)-f\left(\tilde{x}\right)\right|}_{2}^{2}\right]$$

where U is the set of unlabeled data, T represents a set of data augmentations or transformations, and *f* is the model’s prediction function.

**Mixup-Based Methods:**

Mixup trains a model on convex combinations of pairs of examples and their labels. The mixup loss function is:

$$L_{\mathrm{mixup}}=E_{\left(x_{i},y_{i}\right),\left(x_{j},y_{j}\right)}\left[\mathrm{CrossEntropy}(f\left(\lambda x_{i}+\left(1-\lambda \right)x_{j}\right),\lambda y_{i}+\left(1-\lambda \right)y_{j})\right]$$

where $\left(x_{i},y_{i}\right)$ and $\left(x_{j},y_{j}\right)$ are pairs of data points and their labels, and λ is a mixing coefficient typically sampled from a Beta distribution.

**Pseudo-Labeling:**

Pseudo-labeling generates labels for unlabeled data using the model’s predictions and then trains the model on these pseudo-labeled examples. The loss function can be expressed as:

$$L_{\mathrm{pseudo}\text{-}\mathrm{label}}=E_{x∼U}\left[\mathrm{CrossEntropy}(f\left(x\right),\hat{y})\right]$$

where $\hat{y}$ is the pseudo-label generated by the model for an unlabeled data point *x*.

**Teacher-Student Models (Mean Teacher)**:

The mean teacher model, a student network, is trained on labeled data, while its predictions are regularized to be consistent with those of a slowly-updated teacher network. The teacher network’s weights are an exponential moving average (EMA) of the student’s weights. The loss, ensuring that the student’s predictions are close to the teacher’s, is given by:

$$L_{\mathrm{mean}\text{-}\mathrm{teacher}}=E_{x∼U}\left[{\left|f_{\mathrm{student}}\left(x;\theta_{\mathrm{student}}\right)-f_{\mathrm{teacher}}\left(x;\theta_{\mathrm{teacher}}\right)\right|}_{2}^{2}\right]$$

where fstudent and $f_{\mathrm{teacher}}$ are the predictions of the student and teacher models, respectively.

Each approach offers unique strengths and faces specific challenges, shaping their suitability for different types of data and learning tasks. For instance, consistency regularization is robust against input perturbations but may struggle with unrealistic data variations. Mixup methods enhance generalization yet risk creating ambiguous examples. Pseudo-labeling, while straightforward, can suffer from confirmation bias. The mean teacher model ensures stability in learning but requires judicious tuning to maintain the student-teacher alignment.

In this paper, in order to handle the unlabeled data we are inspired by the pseudo-labeling technique. Pseudo-labeling builds upon the general self-training framework [44], where a model goes through multiple rounds of training by leveraging its own past predictions. The key idea behind pseudo-labeling is to use the model’s predictions on the unlabeled data to generate ‘pseudo-labels’/‘pseudo-attributes’ and then use these pseudo-labels to further train the model [43].

Let $D_{L}=\left\{\left(x_{i},y_{i}\right)|i:=1\ldots N_{L}\right\}$, $D_{U}=\left\{\left(x_{i}\right)|i:=1\ldots N_{U}\right\}$ be labeled and unlabeled datasets respectively; we use x and y to denote the vector of instances and their vector of labels. We train a model $f_{\eta }$ with parameters η on the labeled dataset and use $f_{\eta }$ to predict the labels for the unlabeled dataset generating pseudo-labels ${\hat{y}}_{i}$ for each unlabeled example. The training procedure is as follows:

1. Train the model $f_{\eta }\left(x\right)$ on the labeled dataset $D_{L}$ using a supervised loss function, typically cross-entropy (or mean squared error for regression tasks).
  $$L_{s}\left(\eta \right)=-\sum_{i=1}^{N_{L}}\log p_{\eta }\left(y_{i}|x_{i}\right).$$
2. Use the trained model $f_{\eta }\left(x;\theta \right)$ to generate pseudo-labels for the unlabeled dataset $D_{U}$:
  $${\hat{y}}_{j}=\arg\max_{y}p_{\eta }\left(y|x_{j}\right),\;\mathrm{for}\;j=1,\ldots ,N_{U}.$$
3. Create the augmented dataset $D_{a}=D_{L}\cup {\left\{\left(x_{j},{\hat{y}}_{j}\right)\right\}}_{j=1}^{N_{U}}$ by combining the labeled data and the pseudo-labeled data.

Our methodology, though inspired by pseudo-labeling, deviates from its standard implementation. Instead of relying on a pre-trained classifier on labeled data, our approach involves alternating the training of our classifier between labeled and unlabeled data within the same training cycle. This technique allows the model to continuously adapt and learn from both data types simultaneously rather than in distinct phases. A more comprehensive explanation of this methodology is detailed in Section 2.

### 2.2. Variational Autoencoders

Variational Autoencoders (VAEs) [45,46] are a class of deep latent variable models (LVMs). In deep LVMs, the primary objective is to optimize the data likelihood $p_{\theta }\left(x\right)$, where x represents the observed data and θ denotes the model parameters. Formally, the goal is to maximize the marginal likelihood of the observed data, which involves integrating over the latent variables z:

$$p_{\theta }\left(x\right)=\int p_{\theta }\left(x|z\right)p\left(z\right)dz.$$

However, this integration is typically intractable due to the high dimensionality and complexity of the data and model. Instead, variational inference allows this problem to be reframed as an optimization problem by introducing an approximation of the true posterior.

$$q_{\phi }\left(z|x\right)=\underset{q}{arg min}D_{KL}(q_{\phi }\left(z|x\right)∥p_{\theta }\left(z|x\right),$$

that allows a tractable bound on $p_{\theta }\left(x\right)$ to be formed. In particular, VAEs amortize the inference process, that is, approximate $q_{\phi }\left(z|x\right)$ using a inference network allowing scaling to large datasets [45,46]. From the definition of KL divergence, we obtain:

(1) $$\begin{array}{cc} D_{KL}\left(q_{\phi }\left(z|x\right)∥p_{\theta }\left(z|x\right)\right) & =E_{q_{\phi }\left(z|x\right)}\left[\log\frac{q_{\phi }\left(z|x\right)}{p_{\theta }\left(z|x\right)}\right]\\ \; & =E_{q_{\phi }\left(z|x\right)}\left[\log q_{\phi }\left(z|x\right)\right]-E_{q_{\phi }\left(z|x\right)}\left[p_{\theta }\left(z,x\right)\right]+\log p_{\theta }\left(x\right)) \end{array}$$

Rearranging Equation (1), we obtain,

(2) $$\begin{array}{cc} \log p_{\theta }\left(x\right) & =D_{KL}\left(q_{\phi }\left(z|x\right)∥p_{\theta }\left(z|x\right)\right)-E_{q_{\phi }\left(z|x\right)}\left[\log q_{\phi }\left(z|x\right)\right]+E_{q_{\phi }\left(z|x\right)}\left[p_{\theta }\left(z,x\right)\right]\\ \; & \geq -E_{q_{\phi }\left(z|x\right)}\left[\log q_{\phi }\left(z|x\right)\right]+E_{q_{\phi }\left(z|x\right)}\left[p_{\theta }\left(x,z\right)\right]\\ \; & =-E_{q_{\phi }\left(z|x\right)}\left[\log q_{\phi }\left(z|x\right)\right]+E_{q_{\phi }\left(z|x\right)}\left[p_{\theta }\left(z\right)\right]+E_{q_{\phi }\left(z|x\right)}\left[p_{\theta }\left(x|z\right)\right]\\ \; & =E_{q_{\phi }\left(z|x\right)}\left[\log p_{\theta }\left(x|z\right)\right]-D_{KL}\left(q_{\phi }\left(z|x\right)∥p_{\theta }\left(z\right)\right)\\ \; & =L(\theta ,\phi ;x), \end{array}$$

where L is known as the Evidence Lower BOund (ELBO) [45]. In Equation (2), the first term is the reconstruction error (negative), and the second term is the Kullback–Leibler (KL) divergence between the approximate posterior $q_{\phi }\left(z|x\right)$ and the prior distribution $p_{\theta }\left(z\right)$ over the latent variables. Commonly, the prior distribution p(z) is selected as a standard Gaussian distribution, p(z)=N(z|0,I). In the ELBO objective, the reconstruction error measures the model’s ability to reconstruct input data, while the KL divergence regularizes the latent space by encouraging the encoding mechanism to align the latent variables with the prior distribution. This dual aspect of the ELBO, balancing between accurate data reconstruction and adherence to a prior distribution, is central to the effective functioning of Variational Autoencoders.

To optimize the ELBO objective, with respect to the parameters of the model, gradients must be backpropagated through the stochastic sampling process $z∼q_{\phi }\left(z|x\right)$. This is permitted by the reparametrizing z using a differentiable function $g_{\phi }\left(\epsilon ,x\right)$ of a noise variable***ϵ***: $z=g_{\phi }\left(\epsilon ,x\right)$ with ϵ∼p(ϵ). Since the prior distribution p(z) is typically chosen to be a standard Gaussian distribution, p(z)=N(z|0,I), and the inference network a Gaussian distribution with diagonal covariance, $N(z|\mu ,\sigma^{2}I)$, the reparameterization of z simplifies to z=μ+σ⊙ϵ with ϵ∼N(z|0,I).

### 2.3. Conditional VAEs

One of the limitations of VAEs is the lack of control over the data generation process. Conditional Variational Autoencoders (CVAEs), as introduced in [13,47], extend the VAE framework to address this limitation. CVAEs incorporate additional information, y, such as specific attributes or labels, into the generative process, enabling more directed and controlled data generation.

In this context, we focus on CVAEs with conditioning applied solely to the decoder. The conditional decoder takes both the latent variable z and the conditioning variable y as input to reconstruct the data, denoted by $p_{\theta }\left(x|z,y\right)$. The modified objective function becomes:

(3) $$L\left(\theta ,\phi ;x,y\right)=E_{q_{\phi }\left(z|x\right)}\left[\log p_{\theta }\left(x|z,y\right)\right]-D_{\mathrm{KL}}\left(q_{\phi }\left(z|x\right)∥p\left(z\right)\right).$$

By limiting the conditioning only on the decoder, the latent variable z is encouraged to capture the variations in the data that are not explained by the conditioning variable y. This allows the model to learn a representation of the data in the latent space that is disentangled from the conditioning variable. By encoding the data and modifying the conditioning variable y before decoding, it is possible to manipulate the data in a controlled way.

In a semi-supervised setting, the conditioning variable y can represent class labels or attribute values associated with the input data. The labeled data can be used to train the CVAE with both the input data x and the corresponding conditioning variables y. For unlabeled data, where the class labels or attribute values are not available, we can condition on ’pseudo-labels’ or ‘pseudo-attributes’. These are generated by a prediction network, serving as substitutes for actual labels or attributes, thus allowing the conditional model to still operate effectively even in the absence of explicitly labeled data.

In summary, VAEs and their conditional variants offer a powerful framework for learning latent representations of data. By combining VAEs with semi-supervised learning techniques, we can effectively utilize both labeled and unlabeled data, which have the potential to enhance model performance, especially in scenarios where labeled data are limited. However, it is important to note that the extent of performance improvement can vary depending on the specific conditions and the nature of the data.

## 3. BtVAE

We assume that we observe data generated from some joint data distribution p(x,y)=p(x|y)p(y) where x is the random variable that corresponds to the input data; its instantiations, x, take values in some set X. The random variable $y=(y^{1},y^{2},\ldots ,y^{K})$ is the conditioning variable, and its instantiations, y, take values in the set $Y=Y^{1}\times \cdots \times Y^{K}$ where $Y^{k}$ can be a finite value set or the continuous space $R^{D}$, with *D* denoting the dimensionality of the continuous space.

We have access to a labelled and an unlabelled training set, $D_{L}$ and $D_{U}$, respectively, with typically $N_{L}<<N_{U}$. Due to the finite size of these sets, and in particular that of the labelled dataset, the empirical distributions that we can obtain from them might not accurately reflect the true data distribution. Such a situation can be further aggravated due to confounding factors during data collection, which can also bias the empirical training distribution. Of particular interest for us is the setting in which the empirical marginal distribution of the conditioning random variable, $\hat{p}\left(y\right)$, is different from the true marginal p(y).

To clarify, the empirical marginal distribution $\hat{p}\left(y\right)$ might not adequately represent certain subspaces of Y. For discrete conditioning random variables, the empirical marginal distribution $\hat{p}\left(y\right)$ can have zero support for some of the values of Y, i.e., not all possible value combinations under the true prior appear in the $D_{L}$ training set. For continuous conditioning random variables, $\hat{p}\left(y\right)$ might not accurately represent the true density across the entire continuous domain Y. This discrepancy can manifest as under-represented regions or “gaps” within the continuous space.

We have a rather weak requirement that the $\hat{p}\left(y^{k}\right)$ empirical marginal distributions have non-zero support for all values or intervals of $Y^{k}$, i.e., various values or intervals of a given conditioning attribute $y^{k}$ have been seen in the training set. Without this, learning to generate instances with the missing values becomes an insurmountable challenge. Let us denote by $Y_{D}$ the y vector values (or continuous intervals) that appear in the training set and by $Y_{\mathrm{OOD}}=Y-Y_{D}$ the complement, representing non-observed, out of the empirical training distribution, values (or intervals) within Y. To give some concrete examples, if we want to model faces the training set might not contain images of women wearing glasses, but it should contain faces of women and faces with glasses, and we would like to generate faces of women wearing glasses. If we want to model molecules, the training dataset might not contain a range of LogP combined with a particular range molecular weight values; it should nevertheless contain molecules with molecular weight in the desired range as well as molecules in the particular range of LogP values.

We want to learn conditional generative models, p(x|y), that are defined and well behaved for all possible y values under the true prior p(y). This means that we also want to have low reconstruction error and meaningful generations for all y, including those in $Y_{\mathrm{OOD}}$. Additionally, for any pair $\left(x,\hat{y}\right)$ of an input observation x (with its inherent attributes y) and a novel set of attribute value combinations $\hat{y}$, we want to create a modified data point $\hat{x}$ that aligns with the specified attribute values $\hat{y}$. This modified $\hat{x}$ should resemble the original input x in its overall characteristics, except for the changes specified by the attribute values $\hat{y}$ we condition on. In fact, such models trained under the empirical training distribution, $\hat{p}\left(y\right)$, will be undefined in values from $Y_{\mathrm{OOD}}$.

The continuity property of VAEs [48] makes it so that when we decode neighboring latent space points we obtain similar content, allowing us to generate samples that are similar but not identical to the empirical training data distribution. This, taken together with the capability of CVAEs to control the generation process using conditional information, suggests that it is not only possible to generate new samples with desired OOD property value combinations but also to manipulate the properties of OOD data. However, while VAEs demonstrate good reconstrunction quality on OOD data [49], their conditional generation performance on OOD data, and more specifically when the conditioning vector values have not been seen during training, is rather problematic [38,39,40,50]. To address this limitation and generate plausible samples with unseen attribute value combinations, we propose BtVAE, a generative model that brings together CVAE, semi-supervised learning, and back-translation, for OOD conditional generation.

Providing a broad overview, during its training process, BtVAE modifies the input observation by conditioning it on randomly chosen attribute values. This produces a modified input that can have attribute combinations not present in the original training data. Next, the modified input is used to reconstruct the original observation, conditioning on either pseudo-attribute values predicted by the model or the actual attributes when available. By conditioning on randomly chosen attribute values during training, BtVAE learns to handle a wide range of input–attribute combinations, including those that may not be present in the training data. This allows the model to generalize better to OOD data as it becomes more proficient at handling unseen attribute combinations.

More specifically, we leverage the existing labelled and unlabelled training samples to generate in-distribution as well as OOD samples by conditioning the generation step on attribute value combinations that are sampled from some prior distribution that we believe is a better proxy for the non-observed true prior p(y); in the absence of an informed prior, we opt for the uniform distribution $p_{U}\left(y\right)$ over Y. We make sure that the generated samples exhibit the conditioning properties by enforcing appropriate constraints with the help of a learned attribute predictor f:x→y, which maps x instances to their y attribute values. We follow up on this generation step by a back translation step that cancels out the conditioning by using the original attribute values for the labelled data, or pseudo attribute/label information for the unlabelled data produced by the property predictor. This step allows for an indirect evaluation of the quality of the OOD generations; a direct evaluation is not really possible since due to the OOD sampling we do not know what the generated instance should look like, and thus there is no readily available reconstruction error.

Under such a training schema, the learning is exposed to OOD samples, making the conditional generative model defined for all Y values. Moreover, using the learned property predictor to enforce constraints on the conditioning attribute values that were used to generate a particular sample, we make sure that the generative model will indeed generate samples that exhibit the conditioning values.

Overall, our conditional generation model p(x|y) will allow: (i) conditioning on value combinations not present in the training set ($Y_{\mathrm{OOD}}$), (ii) the accurate reconstruction of input data with value combinations in $Y_{\mathrm{OOD}}$, and (iii) attribute manipulation of in-distribution and OOD data with value combinations from $Y_{\mathrm{OOD}}$.

### 3.1. Model

**Figure 1:**
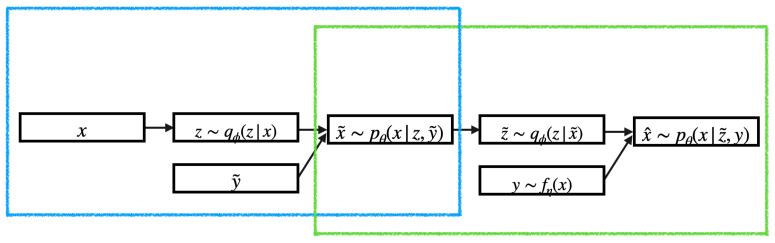
Main architecture of the BtVAE model. The model consists of two components, VAEs with conditional decoders. The first one (blue) modifies the input instance conditioning on randomly sampled attribute values and the second one (green) translates the modified input back to the original input conditioning on the pseudo-attribute values of the original input or the real attribute values if they are available.

The main architectural component of BtVAE is a probabilistic encoder and a conditional probabilistic decoder, a CVAE, which is applied twice in a row, Figure 1. We consider the conditional latent-variable model $p_{\theta }\left(x,z|y\right)=p_{\theta }\left(x|y,z\right)p\left(z\right)$, where x denotes an observation, y represents the associated attribute vector, and z represents the associated latent variable. The marginal p(z)=N(z|0,I) is a prior over the latent variable, and $p_{\theta }\left(x|y,z\right)$ is an exponential family distribution whose natural parameter is a function of z parametrized by θ, e.g., through a neural network. The encoder receives input data points x (labelled or unlabelled) and maps them to their latent representation z. The decoder subsequently takes this latent representation and samples attribute values from the prior, $\tilde{y}∼p_{U}\left(y\right)$, to generate a modified version of the input, $\tilde{x}$. In the case of labelled data with some small probability *p*, we use the original attribute values as conditioning values in order to facilitate the learning of the property predictor since the pairs $\left(\tilde{x},\tilde{y}\right)$ are also used together with the original labelled data for their training. As a result of the way we sample $\tilde{y}$, the generated instance $\tilde{x}$ may possess attribute value combinations that are not present in the training data.

At the generation step, the decoder receives the latent representation z of the original input x and the randomly sampled $\tilde{y}$ and produces a modified $\tilde{x}$.

In a standard VAE, the quality of the reconstruction is typically evaluated by assessing the reconstruction error. However, in our setting this direct evaluation is not possible since we do not have access to the ground truth for the modified instance $\tilde{x}$, and we do not know how such an instance should look like. This learning task can be characterized as unsupervised given that our training set does not contains examples of an input and its associated modified instance $\tilde{x}$ is tailored to the specific attribute values. Thus, we can only indirectly evaluate the quality of $\tilde{x}$. We will do so by ensuring that the generated instance $\tilde{x}$ preserves the content of the original input x and differs only on the conditioning attributes. To ensure the preservation of content, we translate $\tilde{x}$ back to x; such a translation allows us to establish a reconstruction loss. This back-translation procedure situates the model to a pseudo-supervised setting where the modified instances $\tilde{x}$ act as supervised training data for the primary objective.

Back-translation operates as follows: we pass $\tilde{x}$ through the encoder to obtain its latent representation, $\tilde{z}$, and then pass $\tilde{z}$ together with an attribute-value vector y to the decoder to reconstruct the original input x. For the x inputs that are labelled instances, we set y as their true attribute values; for unlabelled instances, we set y to the pseudo-attribute values predicted from the attribute network. The result of back translation is that when we pass into the decoder $\left(\tilde{z},y\right)$, we know what the output should look like, enabling us to train the model to map $\left(\tilde{x},y\right)$ into x. The back-translation process is trained by minimizing the back-translation loss, Equation (4), which is a combination of three terms.

(4) $$\begin{array}{c} L_{bt}=E_{x∼p\left(x\right),y∼p_{U}\left(y\right)}\underset{A}{\underbrace{[E_{q_{\phi }\left(\tilde{z}|\tilde{x}\right)}\log p_{\theta }\left(x|\tilde{z},y\right)}}-\underset{B}{\underbrace{D_{\mathrm{KL}}(\left(q_{\phi }\left(z|x\right)∥p\left(z\right)\right)}}-\underset{C}{\underbrace{D_{\mathrm{KL}}\left(q_{\phi }\left(\tilde{z}|\tilde{x}\right)∥p\left(z\right)\right)]}} \end{array}$$

The A term in the loss is the negative reconstruction cost between the output of the second component of BtVAE and the input of the first one. It can be seen as a cycle consistency loss: $E∥x-\mathrm{Dec}(\mathrm{Enc}\left(\tilde{x}\right),y)∥$ that ensures that the information content of the input x (other than the conditioning variable) will be preserved. Term B penalizes the derivations of the approximate posterior from the latent prior when conditioning on a given input x from the prior, and term C penalizes the derivations of the approximate posterior when conditioning on the modified input $\tilde{x}$.

A trivial way for the model to optimize the back translation loss and to successfully map $\left(\tilde{x},y\right)$ back to the original x would be to disregard the conditioning attribute component $\tilde{y}$ in the first application of the conditioning VAE. In this case, $\tilde{x}$ would merely be a reconstruction of the input data x. To circumvent this issue, we pass the modified input $\tilde{x}$ through the attribute network $f_{\eta }$ and require that it has the target attribute values, $\tilde{y}$, that were used to produce it i.e., $f\left(\tilde{x}\right)\to \tilde{y}$. To learn the attribute network, we optimize the attribute constraint objective to facilitate the learning of the attribute Equation (5),

(5) $$\begin{array}{c} L_{attr}=E_{\tilde{y}∼p_{U}\left(y\right),\tilde{x}∼p_{\theta }\left(\tilde{x}|z,\tilde{y}\right)}\left[l\left(f_{\eta }\left(\tilde{x}\right),\tilde{y}\right)\right], \end{array}$$

where the attributes are binary labels, $f_{\eta }$ is a classifier, and *l* is binary cross entropy, while when the attributes are continuous $f_{\eta }$ is a regressor and *l* is a classical mean squared error (MSE).

To facilitate the learning of the attribute network, we augment its inputs with labeled data. When the attribute values y are available, the attribute constraint objective (Equation (5)) has an extra term that helps the model to learn to predict correctly the corresponding attribute values using the supervised input data. In this case, the final attribute objective is given by Equation (6). This is central since it enables learning the conditional decoder network in a semi-supervised manner.

(6) $$\begin{array}{c} L_{attr}=E_{\left(x,y\right)∼p(x,y)}\left[l\left(f_{\eta }\left(x\right),y\right)\right]+E_{\tilde{y}∼p_{U}\left(y\right),\tilde{x}∼p_{\theta }\left(\tilde{x}|z,\tilde{y}\right)}\left[l\left(f_{\eta }\left(\tilde{x}\right),\tilde{y}\right)\right] \end{array}$$

In this way, the attribute network plays a double role. Beyond ensuring that the modified input $\tilde{x}$ has the desired target attribute values, the attribute network also serves as a pseudo-labeling mechanism, positioning BtVAE as a self-labeling model. This self-labeling property allows BtVAE to generate reasonable pseudo-attribute values for the unlabeled data, which are then used to guide the model during training. To be more precise, in the translation phase, when actual attribute values are absent, BtVAE relies on pseudo-attribute values predicted by the attribute predictor network $f_{\eta }\left(x\right)$ to condition the decoder during the reconstruction of the original observation. As a result, BtVAE can learn to generate better representations of the input data even when the true attribute values are not available. This capability enables the model to handle both labeled and unlabeled data, making it a self-labeling model.

The final objective of the model is obtained by combining the back-translation objective with the attribute constraint regularizer, resulting in:

(7) $$\begin{array}{c} L=L_{bt}+L_{attr} \end{array}$$

We train the model jointly in an end-to-end manner. The pseudo code of our algorithm is shown in Algorithm 1.
**Algorithm 1:** BtVAE: Training Procedure
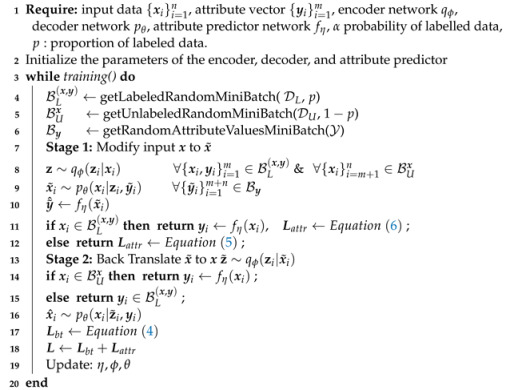


At the beginning of the training, the only reliable information provided to the decoder is the randomly sampled attribute value $\tilde{y}$. Since we explicitly impose the constraint $f_{\eta }\left(\tilde{x}\right)=\tilde{y},\tilde{x}∼p\left(x|\tilde{y}\right)$, this encourages the decoder to use $\tilde{y}$ throughout the entire training. As training progresses, the model employs data augmentation with new inputs $\tilde{x}$ matched to desired attributes y. This leads to generating unique attribute combinations, potentially unseen in the training data, thereby creating out-of-distribution scenarios. By encoding and decoding these new inputs with pseudo-attributes or original labels, the model can manipulate attributes while retaining a clear output vision through back translation. This technique not only evaluates model performance via reconstruction error but also preserves the original input content, x. Thus, the BtVAE model learns to generate novel samples with desired attributes, enhancing its generalization capabilities and handling out-of-distribution scenarios effectively, even with limited labeled data.

### 3.2. Conditioning on Attribute Values Chosen from $P_{U}\left(y\right)$

By expressing the conditional generative model in terms of the underlying joint distribution p(x,y), we can see that the conditional generative model is the ratio of the joint distribution and the marginal distribution of the attributes.

(8) $$\begin{array}{c} \int p\left(x|z,y\right)p\left(z\right)dz=p\left(x|y\right)=\frac{p(x,y)}{p(y)} \end{array}$$

As already discussed above as a result of the finite size of the training set, the empirical marginal distribution $\hat{p}\left(y\right)$ and the empirical joint $\hat{p}\left(x,y\right)$ may deviate from the respective true distributions. Nevertheless, the conditional generative model is shared across the in-distribution and the OOD data. Depending on the prior from which we choose to sample the y values, we obtain different joint distributions. This highlights the importance of conditioning on randomly selected attribute values. By doing so, we are able to sample new synthetic data during the training by using attribute value combinations that do not exist in the in-distribution data. This strategy enables us to learn a conditional generative model that is defined and applicable beyond the empirical marginal distribution.

## 4. Experimental Setup

We evaluate our model on two conditional generation tasks. In the first task, we learn to generate samples from datasets featuring independent controllable factors. In the second task, we learn to generate MNIST digits conditioning on various continuous characteristics. Our goals are three-fold: we would like to examine the ability of our model to (1) generate data conditioning on attribute value combinations absent in the training set ($Y_{\mathrm{OOD}}$), (2) reconstruct data even with attribute value combinations from $Y_{\mathrm{OOD}}$ accurately, and (3) manipulate attributes of in-distribution and OOD data with value combinations from $Y_{\mathrm{OOD}}$.

For our experimental setup, we employ custom controlled datasets, following the procedure of [38,39,40]. This allows us to assess the model’s systematic generalization capabilities to unseen attribute value combinations. We utilize the dSprites [51], Shapes3D [52], and MNIST [53] datasets. Both dSprites and Shapes3D consist of images generated from a set of discrete factors of variation following a deterministic generative model. Each dataset is designed to realize all possible combinations of factors of variation in a corresponding images. These factors are easily interpretable and vary independently, making dSprites and Shapes3D ideal for studying combinatorial generalization. Furthermore, we employ the MNIST dataset, where, with hand-designed functions, we define two statistical descriptors. This allows us to examine how our model performs in scenarios involving OOD continuous attribute value combinations.

### 4.1. Custom Datasets

The efficacy of our method is evaluated on three datasets. We use two datasets featuring independent controllable factors, dSprites [51] and Shapes3D [52], and one dataset with continuous characteristics, MNIST [53]. dSprites comprises 2D shapes generated using six distinct latent factors: color, shape, scale, rotation, and the x and y positions of a sprite. The attributes of interest on this dataset are the x and y positions of a sprite. Shapes3D includes 3D colored images of objects in a room, created from six separate factors: floor color, wall color, object color, object size, object type, and camera azimuth. The attributes that we want to control on the Shapes3D dataset are the floor and wall color. Given that both the dSprites and Shapes3D datasets are generated using distinct latent factors, we consciously chose to manipulate only two attributes. This decision offers latitude for latent variables to manage residual information, enabling us to learn a meaningful latent representation. Table 1 presents the conditioning attributes at each dataset we experiment on.

The MNIST dataset consists of handwritten digits (0–9), each varying in stroke thickness and tilt. We decided to exclude the ‘digit ID’ attribute from the conditioning set due to its strong informativeness. Instead, using hand-designed functions, we defined simple statistical descriptors representing stroke width and digit tilt, enabling their use in our experimental framework.

To examine combinatorial generalization, we follow [38,39,40] and create separate splits for training sets $D_{train}$ and test sets $D_{test}$. We exclude certain combinations of generative factors for dSprites and Shapes3D data, as well as a subset of the MNIST dataset from the training data.

To create the training/test split, all examples with combinations of a subset of attribute values are excluded from the training set and added to the test set. Thus, an example of a dataset may consist of a training set of all combinations where $\left[g_{1}>0.5,g_{2}>0.5\right]$ have been excluded from the training set and have been added to the test set. It should be noted that models trained on such datasets would encounter instances where $\left[g_{1}>0.5\right]$ and instances where $\left[g_{2}>0.5\right]$ but would never be trained on an example where both conditions were simultaneously true. Following this approach, we constructed the training/test sets for each dataset in the following manner (see also Table 2, Table 3 and Table 4):

- dSprites: Images with xPos>0.5 and yPos>0.5 are excluded from the training set. Consequently, a shape never appears in the image’s top-right corner but is present in the bottom-left and top-left corners.
- Shapes3D: Images meeting the criteria [floorColor<0.3] and [wallColor>0.5] are removed from the training data. As a result, floor colors in the initial third of the HSV spectrum (red, orange, etc.) and wall colors in the latter half of the HSV spectrum (blue, purple, etc.) are not present in the training set. This means that combinations of floor colors such as red and orange are observed only with wall colors like red and orange.
- MNIST: Images of digits 7 and 2 with −0.9<StrokeWidth<1.5 and −1<Tilt<0.5 are excluded from the training set. Neither digit 7 nor 2 appears with StrokeWidth equal to 1 and Tilt equal to 0, but digit 8 might exhibit this combination of property values.

**Table 2:** dSprites data splits.

	Shape	Scale	Orientation	Position X	Position Y
Training	Any	Any	Any	y≤0.5	y≤0.5
Test	Any	Any	Any	y>0.5	y>0.5

**Table 3:** Shapes3D data splits.

	Object Color	Scale	Object	Orientation	Floor Color	Wall Color
Training	Any	Any	Any	Any	y≥0.3	y≤0.5
Test	Any	Any	Any	Any	y<0.3	y<0.5

**Table 4:** MNIST data splits.

	Digits	Stock Width	Tilt
Training	2,7	y<−0.9∪y>1.5	y<−1∪y>0.5
	1,3,4,5,6,8,9	Any	Any
Test	2,7	−0.9<y<1.5	−1<y<0.5
	1,3,4,5,6,8,9	Any	Any

The training set contains only in-domain data, while the test set, if fully OOD, means that $D_{test}=\left\{\left(x_{i},y_{i}\right)|i:=1\ldots N_{test}\right\},y\in Y_{OOD}$. Moreover, during the training phase, only 10% of the label information is accessible, while at test time we only use unlabeled data.

### 4.2. Baselines

BtVAE is based on the framework of the conditional VAE. To assess its effectiveness in addressing OOD reconstruction, attribute manipulation, and conditional generation, we compare it against a conditional VAE as a baseline. Furthermore, we compare our model with the CsVAE [54], PCVAE, [55] and MSP [56]. CsVAE, like BtVAAE, is based on a conditional VAE model but uses two latent variables to separate the information correlated with the attributes y into a pre-defined subspace. This separation is achieved by minimizing the mutual information between z and y, and it results in better control over the generative process. PCVAE also utilizes a conditional VAE model and employs two latent variables, similarly to [13,54], including a property prediction network to predict the continuous attributes from the latent variable. This property prediction network maps property values to corresponding latent variables. Such a design helps the model to learn a more accurate representation of the attributes, thereby improving the controllability of sample generation. The MSP model, on the other hand, uses orthogonal matrix projection onto subspaces to factor out the information about the attributes of interest y from the latent variable z. The advantage of this orthogonal matrix projection is that it leads to a more structured and organized latent space, where the different dimensions are distinctly separated and aligned with specific attributes or features. This enables the model to manipulate the latent space, allowing for attribute manipulation of the input data. The CsVAE, PCVAE, and MSP models allow for more precise control over the latent representation, which could have potential implications in OOD scenarios.

All of the mentioned models are supervised, where the attributes are used to enforce invertible mutual dependence between them and the latent variables. To manage unlabeled data, we integrated an attribute predictor network. Our evaluation aims to investigate the ability of these models to reconstruct OOD data, manipulate attributes of unseen data, and generate samples conditioned on previously non-seen attribute value combinations.

### 4.3. Evaluation

We adopt the $R^{2}$ score based on the mean squared error (MSE), the coefficient of determination, as our primary evaluation metric [40,57]. This score measures how well the model can predict attribute values in the presence of OOD data. We define the $R^{2}$ score per attribute $y_{i}$ as follows:

(9) $$\begin{array}{ccccc} R_{i}^{2}=1-\frac{MSE_{i}}{\sigma_{i}^{2}} & \; & \mathrm{with} & \; & MSE_{j}=E_{\left(x,y\right)∼D_{test}}\left[{\left(y_{j}-f_{j}\left(x\right)\right)}^{2}\right] \end{array}$$

Here, $\sigma_{i}^{2}$ denotes the variance of each attribute across the entire dataset. Under this score, $R^{2}=1$ indicates a perfect fit and a value close to zero, $R^{2}=0$, can be interpreted as random guessing since the MSE would be approximately equal to the variance per attribute. Since the $R^{2}$ score can be negative while $R^{2}=0$ indicates random guessing, we clip all negative $R^{2}$ scores to zero [40,57]. The function $f_{j}$ refers to a multi-layer perceptron (MLP) network trained on the entire dataset. The purpose of this network is to predict the attribute values based on the input data.

Additionally, we utilize the mean squared error (MSE), specifically for the conditioning attributes, as a supplementary evaluation metric. This approach aids in gaining a comprehensive understanding of our model’s performance. The MSE is formulated as follows:

(10) $$MSE=\frac{1}{n}\sum_{i=1}^{n}{\left(y_{i}-{\hat{y}}_{i}\right)}^{2}$$

Here, $y_{i}$ represents the conditioning attribute values and ${\hat{y}}_{i}$ denotes the predicted values by the model, with the summation extending over all *n* data points in the test set (OOD). By quantifying the average squared deviation between the predicted and conditioning values, the MSE offers a clear indication of the average error magnitude, which is crucial for assessing the precision of the model’s predictions in the context of attribute-based conditioning.

Utilizing both the $R^{2}$ score and MSE allows us to evaluate our model’s performance in terms of both fitting and predicting attributes. In the context of attribute manipulation and conditional generation experiments, the $R^{2}$ score and MSE are computed between the predicted attribute values and the desired attribute values used for conditioning. This dual metric approach enables us to thoroughly assess the model’s capability in generating outputs with desired characteristics and to evaluate the precision of these predictions. In the case of reconstructions, the $R^{2}$ score and MSE are computed between the predicted attribute values and the respective ground truths, evaluating the model’s ability to preserve OOD attribute value combinations while reconstructing OOD data.

In our model, the objective is to generate data with desired OOD attribute value combinations while preserving the remaining characteristics unaltered. For this purpose, besides the $R^{2}$ score, we utilize the Mean Absolute Error (MAE) criterion,

(11) $$\begin{array}{c} MAE=\frac{1}{n}\sum_{i=1}^{n}\left|y_{i}-{\hat{y}}_{i}\right| \end{array}$$

to quantitatively assess the model’s capability in maintaining the characteristics of the inputs we wish to remain unchanged. We train a regression MLP on the entire dataset to predict the attribute values of a given input. During testing, we compute the Attribute-wise MAE between the predicted attribute values of the generated outputs and the corresponding ground truth of the inputs. Specifically, for the dSprites dataset, we compute the MAE for the shape of the object; for the Shapes3D dataset, we compute it for the object and the object color; and for the MNIST dataset, we compute it for the digit ID.

### 4.4. Model Architectures

Based on the description of the implementation of the proposed objective, there are three components, namely, the encoder to model $q_{\phi }\left(z|x\right)$, decoder to model $p_{\theta }\left(x|z,y\right)$, and attribute predictor network to model $f_{\eta }\left(y|x\right)$. Our architectures consist of convolutional layers with ReLU activations followed by fully connected layers with ReLU activations, similar to the design in [55]. We used the ADAM optimizer with a learning rate of 0.0005 and a mini-batch size of 256. For a fair comparison, all the baselines use the same network architecture.

### 4.5. Experiments and Results

The primary objective is to elucidate the performance of the proposed model on the defined OOD test sets split, as delineated in Section 4.1. OOD data are characterized by attribute value combinations absent during training, represented as $D_{\mathrm{test}}=\left\{\left(x_{i},y_{i}\right)|i:=1\ldots N_{\mathrm{test}}\right\},y\in Y_{\mathrm{OOD}}$. All of the models are trained utilizing a mere 10% of the labeled data.

The evaluation framework comprises three aspects: (1) reconstructing OOD data from the designated test dataset split $D_{\mathrm{test}}$ in both a supervised and an unsupervised manner; (2) manipulating attributes of the data in both an in-distribution and OOD manner; and (3) generating samples conditioned on y properties from $Y_{OOD}$, representing unencountered property combinations during training.

To benchmark the models’ efficacy in utilizing the conditioning values, we compute the $R^{2}$-score, indicative of the coefficient of determination, on the respective entire test set (see Section 4.3). Furthermore, for the tasks of reconstruction and attribute manipulation, it is essential to ascertain whether the model retains the characteristics of the input data intended to remain unchanged. This verification is effectuated by computing the MAE score. As elaborated in Section 4.3 for the entire test dataset, we compute the MAE score for the shape of the object, for the dSprites dataset; for the object and the object color for the Shapes3D dataset; and for the digit ID, for the MNIST dataset.

#### 4.5.1. OOD Reconstruction

In the OOD reconstruction task, we aim to investigate the ability of our model to reconstruct OOD data, meaning data with attribute value combinations in $Y_{\mathrm{OOD}}$. We examine our model’s ability in two scenarios. First, we consider a supervised setting where the corresponding attributes are available; in this instance, we condition the decoder on the actual attribute values, $\left(x,y\right)\in D_{L}$. Then, we examine the performance of our model in the unsupervised setting wherein the attributes are unavailable. In this scenario, we use pseudo-attributes for the values of the conditioning attributes, which are predicted using the attribute predictor network, $y=f_{\eta }\left(x\right)$. We compute the $R^{2}$ score of the models using the conditioning attributes.

**Figure 2:**
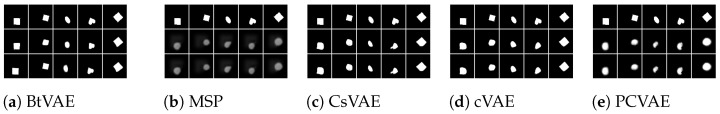
dSprited OOD reconstructions.

**Figure 3:**
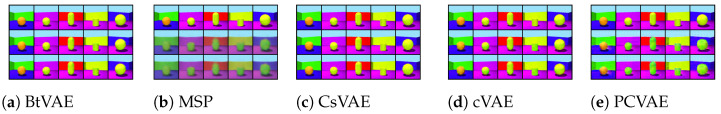
Shapes3D OOD reconstructions.

**Figure 4:**
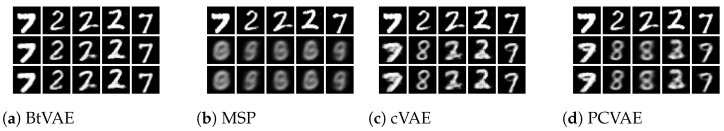
MNIST OOD reconstructions.

Figure 2, Figure 3 and Figure 4 illustrate the OOD reconstructions of the dSprites, Shapes3D, and MNIST datasets, respectively. The first row presents the OOD data targeted for reconstruction, the second row demonstrates reconstruction of the OOD data conditioning on corresponding attribute values (supervised reconstructions), and the third row shows unsupervised reconstruction conditioning on pseudo-attribute values, predicted from the attribute network, $f_{\eta }$. In Table 5, we report the $R^{2}$ score for the three datasets for both the supervised and unsupervised reconstructions. The $R^{2}$-score for each model is almost the same in supervised and unsupervised settings, indicating that the attribute predictor network, $f_{\eta }$, can successfully predict the pseudo-attribute values. This can also be confirmed visually by looking at the second and third lines in Figure 2, Figure 3 and Figure 4. Reconstructing the input data conditioning on the real attribute values, y, (the second line) instead of the predicted pseudo-attribute values, $y=f_{\eta }\left(x\right)$, (the third line) does not improve the reconstructions of the OOD input data (the first line). In some cases, such as the MSP model with the Shapes3D dataset, the $R^{2}$-score of the of the unsupervised case is greater than that in the supervised case. However, the difference between them is very small, which indicates once again that the attribute predictor network can successfully predict the pseudo-attribute values.

In Figure 2, Figure 3 and Figure 4, we can also see that BtVAE successfully reconstructs OOD data for other datasets, in contrast to other models that struggle to reconstruct the correct shape in the case of the dSprites dataset and the correct digit in the MNIST dataset. This is also confirmed in Table 6, where we report the MAE score for the shape of the object, for the dSprites dataset; the MAE score for the object and the object color, for the Shapes3D dataset; and the MAE score for the digit ID, for the MNIST dataset.

For the Shapes3D dataset, the OOD reconstruction is successful for all the models expect MSP (Figure 3). The hight $R^{2}$-score (Table 5) and the low MSE (Table 7) indicate the models’ ability to effectively utilize conditioning attributes, floor color, and wall color during the reconstruction process, even though the attribute combinations are absent from the training data, while preserving the object shape and color of the input data. Moreover, the low MAE (Table 6) indicates that the models also preserve the rest characteristic of the data.

#### 4.5.2. Attribute Manipulation

In the attribute manipulation task, we aim to manipulate the input data in a controllable way with the desired property values. Given an initial OOD input from the $D_{test}$ with certain y property values, the objective is to modify these property values to new desired values. These modifications are conducted in two distinct manners: in the in-distribution manner, where the new value combinations have already been seen in the training set, and in the OOD manner, where the value combinations were not present in the training set. Using the latent representation of an input image, we generate eight different modified images. Among these, four are intended to possess in-distribution attribute value combinations, while we want the remaining four to have OOD attribute value combinations. For all the generated images, we manipulate all the conditioning attributes concurrently, maintaining consistent attribute value combinations across all images and across all different methods. For the dSprite dataset, we manipulate the Position X and Position Y attributes; for the Shapes3D dataset, the floor color and wall color; and for the MNIST dataset, the stroke width and the tilt.

**Figure 5:**
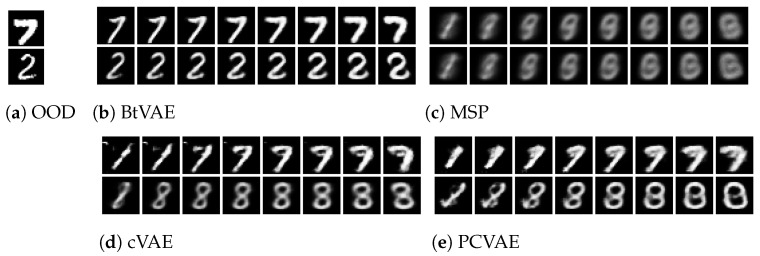
MNIST: attribute manipulation of the OOD inputs (**a**), in in-distribution manner (**b**–**e**): (first–fourth column) and in out-of-distribution (**b**–**e**): (5th–eighth column) manner.

**Figure 6:**
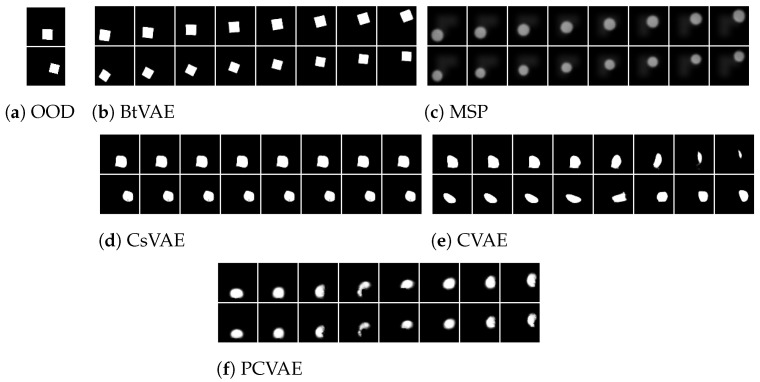
dSprites OOD attribute manipulation: using the latent representation of the images in Figure 6, we manipulate their attribute values by conditioning on in-distribution values (first–fourth columns) and on OOD values (5th–eighth columns).

**Figure 7:**
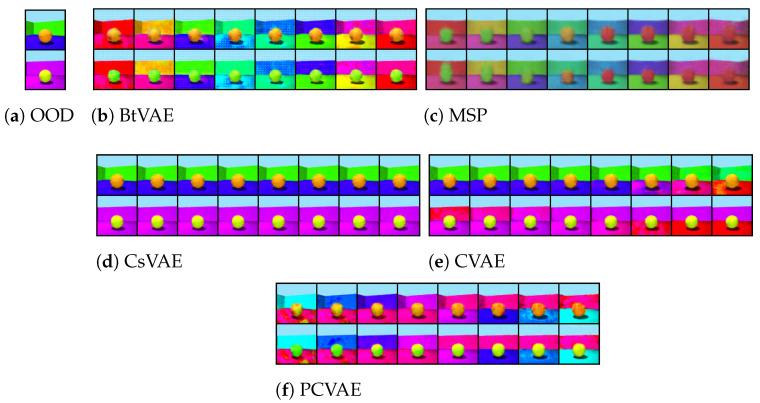
Shapes3D ODD attribute manipulation: using the latent representation of the inputs in (**a**) we manipulate them by conditioning on eight different the floor and wall color combinations. In first–fourth columns of (**b**–**f**), we condition on in-distribution combinations and in out-of-distribution and in fifth–eighth columns on OOD combinations.

In Figure 5, Figure 6 and Figure 7, we present the results of the various methods across the different datasets. Within each figure, subfigure (a) displays the original instance whose attribute we intend to modify; subfigures (b) through (e/f) correspond to the different methods. The first four columns in each of these subfigures illustrate the in-distribution attribute manipulation results, while the latter four columns provide the OOD manipulation results.

Table 8, Table 9 and Table 10 report the $R^{2}$-score and the MSE for the conditioning attributes to evaluate the ability of different methods to manipulate them effectively. Moreover, the MAE score for the characteristics that are intended to remain unchanged is also reported (the shape of the object for the dSprites dataset, the object and the object color for the Shapes3D dataset, and the digit ID for the MNIST dataset). Both scores were computed across the entire dataset while conditioning on OOD attribute value combinations.

BtVAE effectively manipulates the attributes, even for combinations not encountered during training, achieving higher $R^{2}$ scores and lower MSE compared to the other methods while maintaining low MAE for the characteristics we choose not to manipulate across all datasets (Table 8, Table 9 and Table 10). For instance, in the dSprite dataset, Figure 6, BtVAE stands as the sole method that retains the shape of the initial inputs while successfully altering the Position X and Position Y attributes, as seen in Figure 6b. This capability of BtVAE to preserve the shape of the initial input while addressing the desired properties is a result of the back-translation procedure, which guarantees content preservation. On the contrary, MSP and PCVAE, although able to manipulate the Position X and Position Y, are unable to preserve the shape of the input images, as illustrated in Figure 6c and Figure 6f, respectively. Moreover, CsVAE and CVAE, seen in Figure 6d and Figure 6f, respectively, ignore the conditioning attributes and fail to maintain the shape of the input images.

In the MNIST dataset, we aim to manipulate the stock width and Tilde attributes simultaneously while preserving the Digit ID. Precisely, we encode an input image; Figure 5a tilt combinations should generate images with the desired value combinations while preserving the Digit ID of the inout image.

Figure 5 illustrates that all models, except BtVAE, encounter difficulties in the OOD setting, often generating a digit resembling the desired one while maintaining the given attribute value combination. As we can see in Figure 5c–e, MSP, CVAE, and PCVAE fail to preserve the digit of the input image. For instance, digit 2 is substituted with digit 8. In contrast, BtVAE, as shown in Figure 5b, maintains the Digit ID of the input images. This observation is further corroborated by the MAE score for the Digit ID across different methods, as seen in Table 8. BtVAE exhibits the lowest MAE, indicating a preservation of the Digit ID. Concurrently, BtVAE attains the highest $R^{2}$-score and the lowest MSE, signifying its effectiveness in accommodating the desired values.

Though BtVAE is built upon the CVAE, it outperforms CVAE due to two key factors. Firstly, the random sampling procedure employed during training empowers BtVAE with the ability to generate novel samples with desired properties, encompassing OOD combinations. Secondly, the back-translation procedure contributes to the preservation of the original input’s content, making it feasible to manipulate the data by allowing the editing of desired properties while maintaining their content.

#### 4.5.3. Conditional OOD Generation

To evaluate the conditional OOD generation performance of the different methods, we first sample 2k latent variables z from the prior distribution p(z), pairing each one with OOD value combinations y randomly sampled from the test set, followed by decoding the concatenated pair (z,y). To quantify the quality of the conditional generations, we measure the attribute values of these generated samples and compute the $R^{2}$-score to ascertain the correlation between these values and the attribute value combinations used for conditioning the generation. This score essentially provides insight into how well the generated samples align with the desired OOD attribute value combinations specified during the conditional generation process.

In Table 11 and Table 12, we report the $R^{2}$-score ANd MSE evaluating the OOD conditional generation capability of the different methods on dSprites, Shapes3D, and MNIST datasets, respectively. As we can see, BtVAE has a considerably higher $R^{2}$-score and lower MSE on all the datasets compared to the other methods.

**Figure 8:**
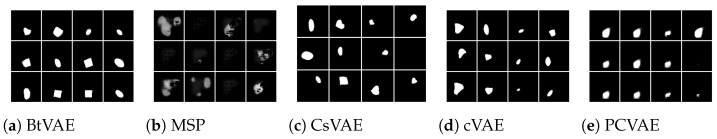
dSprites samples conditioned on OOD attribute values. All of the samples are generated by conditioning on the same xPosition and yPosition values.

**Figure 9:**
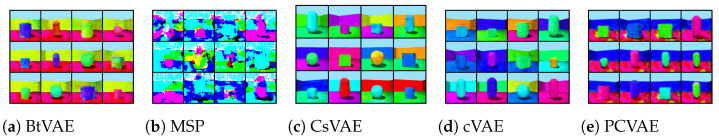
Shapes3D samples conditioned on OOD attribute values. All the samples are generated conditioning on the same floor and wall color.

**Figure 10:**
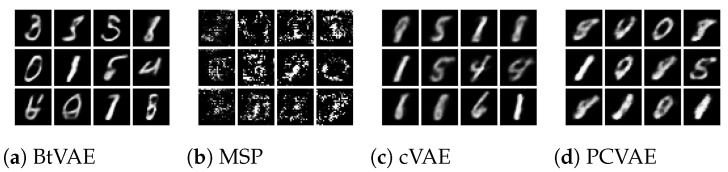
MNIST samples conditioned on OOD attribute values. All the samples are generated conditioning on the same StrokeWidth and Tile values.

In Figure 8, Figure 9 and Figure 10, we display 12 conditional samples generated by each method using the dSprites, Shapes3D, and MNIST datasets, respectively. To facilitate a clearer understanding of whether the conditioning values are being utilized, we generate 12 samples for each, all conditioned on identical combinations of attribute values. For instance, with the Shapes3D dataset, Figure 9, we sample 12 different z from the prior distribution p(z), and each one is concatenated with the same attribute values—specifically, ‘red floor’ and ‘yellow wall’. This concatenation forms the input to the decoder of our model, aiming to generate samples that consistently display the ‘red floor’ and ‘yellow wall’ but differ in other attributes like object shapes and colors.

As illustrated in Figure 8a, Figure 9a, and Figure 10a, BtVAE efficiently leverages the target attributes while concurrently generating a varied array of shapes, colored objects, and digits for the dSprites, Shapes3D, and MNIST datasets, respectively. This underscores the successful learning of the latent representations and that the model is disentangling the latent representations from the conditioning attributes. Regarding the MNIST dataset, it is worth mentioning that, besides BtVAE, both PCVAE and CVAE also achieve the task of conditional generation based on OOD attribute values as seen in Figure 10, albeit with lower $R^{2}$-scores as recorded in Table 11 than BtVAE. This observation suggests that these models, particularly PCVAE, also possess the ability to effectively harness target properties while generating a diverse range of digits conditioned on previously unseen combinations of attributes.

For the dSprites dataset, BtVAE generates a variety of shapes with varying scales and orientations as depicted in Figure 8a while ensuring that all the samples adhere to the desired Position X and Position Y values with the highest $R^{2}$-score ($R^{2}=0.998$) and lowest MSE (MSE =0.0001). This not only signifies that the proposed method employs the conditioning properties accurately but also demonstrates the successful learning of a meaningful latent representation. In contrast, PCVAE, showcased in Figure 8e, albeit utilizing the OOD attribute value combinations, fails to learn a meaningful latent representation, which results in the generation of poorly shaped samples. On the other hand, MSP, CsVAE, and CVAE, as highlighted in Figure 8b–d, encounter difficulties both in learning a good latent space and in accounting for the desired properties. Similar observations can be made for the Shapes3D dataset, as we can see in Figure 9. Here, BtVAE, Figure 9a, stands out as the only method that heeds the conditioning OOD attribute value combinations, generating samples across a variety of shapes and colors. In contrast, for this dataset, PCVAE—depicted in Figure 9e—manages to generate samples that consider only one of the conditioning attributes, struggling to produce samples when conditioned on OOD value combinations, evidenced by an $R^{2}$-score of 0.38 as opposed to BtVAE’s 0.99.

## 5. Conclusions

In this paper, we have introduced BtVAE, a self-labeling model based on Conditional Variational Autoencoders, designed to handle out-of-distribution (OOD) data effectively. By leveraging back-translation and conditioning on randomly chosen attribute values during training, BtVAE learns to handle a diverse range of input–attribute pairs that may not be present in the training data, thus enhancing its capability to handle OOD data. Moreover, due to the back translation procedure BtVAE preserves the content of the input data while manipulating their attribute values. We demonstrated its effectiveness on different datasets and compared its performance with other state-of-the-art models, such as CsVAE, PCVAE, and MSP. Our evaluation showed that BtVAE is capable of reconstructing OOD data, manipulating attributes of unseen data, and generating samples conditioned on non-seen attribute value combinations regardless of the dataset utilized. Contrarily, the other models exhibited limitations, either failing to adequately handle OOD attribute values or struggling to preserve the original content during attribute manipulation. Furthermore, BtVAE’s capability to leverage target properties while generating a diverse range of outputs is prominently showcased, along with it capability to preserve the content of an initial input, underlining its potential for real-world applications that require both attribute-specific manipulations and the generation of conditioned, diverse samples.

For future work, we aim to extend the BtVAE model to handle more complex datasets, such as molecular datasets, and investigate its applicability in other domains, like drug design. Additionally, we plan to explore the integration of other self-labeling techniques and unsupervised learning methods to further improve the model’s performance on OOD data. Lastly, we intend to investigate the potential applications of our model in various fields, including anomaly detection, data augmentation, and domain adaptation, where handling OOD data is of paramount importance. 

## Figures and Tables

**Table 1:** Conditioning attributes at each dataset.

dSprites	Shapes3D	MNIST
Position X, Position Y	Floor color, Floor color	Stock width, Tilt

**Table 5:** $R^{2}$-score evaluating the reconstruction capability of the various models on dSprites, Shapes3D, and MNIST datasets in both supervised and unsupervised settings.

	dSprites		Shapes3D		MNIST	
	Sup.	Unsup.	Sup.	Unsup.	Sup.	Unsup.
BtVAE	0.945	0.932	0.999	0.999	0.963	0.956
MSP	0.287	0.216	0.437	0.442	0.858	0.851
CsVAE	0.375	0.361	0.999	0.999	-	-
PCVAE	0.825	0.812	0.999	0.999	0.941	0.941
cVAE	0.8	0.665	0.999	0.999	0.946	0.933

**Table 6:** MAE score for the shape of the object, for the dSprites dataset; the object and the object color, for the Shapes3D dataset; and the digit ID, for the MNIST dataset of the reconstructed data in the unsupervised setting.

	dSprites	Shapes3D		MNIST
	Shape	Object.	Obj. Color	Digit ID
BtVAE	0.080	0.026	0.025	0.0928
MSP	0.340	0.180	0.790	2.230
CsVAE	0.112	0.023	0.028	-
PCVAE	0.193	0.062	0.095	0.846
cVAE	0.123	0.0258	0.0298	1.054

**Table 7:** MSE score evaluating the reconstruction capability of the various models on dSprites, Shapes3D, and MNIST datasets in the unsupervised settings.

	dSprites	Shapes3D	MNIST
BtVAE	0.015	0.00005	0.041
MSP	0.061	0.045	0.135
CsVAE	0.053	0.00008	-
PCVAE	0.028	0.00006	0.051
cVAE	0.032	0.00008	0.063

**Table 8:** MNIST attribute manipulation: $R^{2}$-score for the conditioning attributes, stock width, and title; and MAE score for the Digit ID.

	$R^{2}$	MSE	MAE
BtVAE	0.943	0.043	0.19
MSP	0.753	0.134	2.23
PCVAE	0.832	0.069	0.94
cVAE	0.921	0.061	1.05

**Table 9:** dSprites attribute manipulation: $R^{2}$-score for the conditioning attributes, *X*-Position, and *Y*-Position; and MAE score for the shape.

	$R^{2}$	MSE	MAE
BtVAE	0.945	0.004	0.016
MSP	0.703	0.052	0.342
CsVAE	0.148	0.643	0.090
PCVAE	0.506	0.067	0.115
cVAE	0.405	0.083	0.124

**Table 10:** Shapes3D attribute manipulation: $R^{2}$-score for the conditioning attributes, floor, and wall color; and MAE score for the object shape and object color.

	$R^{2}$	MSE	MAE	
			Obj.	Obj. Color
BtVAE	0.94	0.00006	0.024	0.032
MSP	0.453	0.044	0.194	0.0785
CsVAE	0	0.103	0.023	0.028
PCVAE	0.13	0.092	0.025	0.029
cVAE	0.267	0.059	0.026	0.013

**Table 11:** $R^{2}$-score evaluating the OOD conditional generation capability of the different methods on dSprites, Shapes3D, and MNIST datasets.

	dSprites	Shapes3D	MNIST
BtVAE	0.998	0.99	0.941
MSP	0.06	0	0
CsVAE	0	0.19	0
PCVAE	0.488	0.388	0.838
cVAE	0.325	0	0.218

**Table 12:** MSE score evaluating the OOD conditional generation capability of the different methods on dSprites, Shapes3D, and MNIST datasets.

	dSprites	Shapes3D	MNIST
BtVAE	0.0001	0.0021	0.051
MSP	0.107	0.149	1.23
CsVAE	0.151	0.064	2.75
PCVAE	0.081	0.045	0.077
cVAE	0.057	0.128	0.154

## Data Availability

Data are contained within the article.
